# Understanding and training for the impact of large language models and artificial intelligence in healthcare practice: a narrative review

**DOI:** 10.1186/s12909-024-06048-z

**Published:** 2024-10-07

**Authors:** Liam G. McCoy, Faye Yu Ci Ng, Christopher M. Sauer, Katelyn Edelwina Yap Legaspi, Bhav Jain, Jack Gallifant, Michael McClurkin, Alessandro Hammond, Deirdre Goode, Judy Gichoya, Leo Anthony Celi

**Affiliations:** 1 Faculty of Medicine and Dentistry, University of Alberta, Edmonton, AB Canada; 2 Yong Loo Lin School of Medicine, National University of Singapore, Singapore, Singapore; 3 Institute for Artificial Intelligence in Medicine, University Hospital Essen, Essen, Germany; 4 Laboratory for Computational Physiology, Massachusetts Institute of Technology, Cambridge, MA USA; 5 Department of Biostatistics, Harvard T.H. Chan School of Public Health, Boston, MA USA; 6 University of the Philippines Manila College of Medicine, Ermita Manila, Philippines; 7 Department of Critical Care, Guy’s and St Thomas’ NHS Foundation Trust, London, UK; 8 Department of Psychiatry, Yale School of Medicine, New Haven, CT USA; 9 Harvard University, Cambridge, MA USA; 10 Division of Hematology/Oncology, Department of Pediatric Oncology, Boston Children’s Hospital, Boston, MA USA; 11 Department of Emergency Medicine, Beth Israel Deaconess Medical Center, Boston, MA USA; 12 Department of Radiology, Emory School of Medicine, Atlanta, GA USA

**Keywords:** Language Model, Medical Education, Technology, Ethics

## Abstract

Reports of Large Language Models (LLMs) passing board examinations have spurred medical enthusiasm for their clinical integration. Through a narrative review, we reflect upon the skill shifts necessary for clinicians to succeed in an LLM-enabled world, achieving benefits while minimizing risks. We suggest how medical education must evolve to prepare clinicians capable of navigating human-AI systems.

## Introduction

Recent advances in large language model (LLM) technology have raised excitement about their possible role in clinical practice. Even general-purpose models such as OpenAI’s ChatGPT have shown significant promise in these applications, as perhaps best captured in a highly publicized paper wherein the model successfully passed the United States Medical Licensing Examinations (USMLE) [1]. Results have been even more impressive for domain-specific models, such as Google’s Med-PaLM2 or GatorTron [2], which answered a wide range of medical questions at an expert level [3]. These striking results raise an important question: are medical education systems adequately preparing the next generation of clinicians to work alongside these models?

Within this article, we offer a brief outline of recent LLM progress as it relates to healthcare, and seek to envision how these models—alongside other artificial intelligence (AI) technologies-–may shift the nature of clinical practice. We critically examine the clinical competencies which will grow and diminish in value in this context, and place a particular emphasis on the role of clinicians in addressing the safety, ethics, and bias vulnerabilities present with such models. While we emphasize LLMs as particularly transformative tools, we seek to situate them in the broader health informatic landscape, and offer an overview of broad informatics, AI-specific, and LLM-specific skills. Finally, we examine the existing context of medical education, and offer an outline of changes which will be necessary to prepare the healthcare workforce to best take advantage of opportunities afforded by these tools while avoiding their associated pitfalls.

### Envisioning the potential of language models in healthcare

Since Google’s invention of the Transformer architecture in 2017[4], the LLM field has rapidly advanced in both capability and complexity. At the most fundamental level, the goal of these models is to predict and produce the next token (word or part of word) in a string of text. However, the resulting models demonstrate significant emergent behaviors and the ability to interpret a wide range of inputs to produce complex, nuanced, and contextually-appropriate text. Thus, LLMs have shown robust results in natural language processing applications as a deep learning algorithm for general-purpose language and textual interpretation.

At the clinical level, these models have demonstrated the ability to answer complex, context-specific medical knowledge questions accurately [1, 5, 6], as well as to structure and summarize clinical data [7]. It is not difficult therefore, to envision a future wherein many aspects of day-to-day clinical practice are LLM-facilitated. At the research level, LLMs hold promise to aid in understanding and generating healthcare insights from clinical records and other health-adjacent databases by recognizing, summarizing, translating, predicting and generating text from massive training datasets [5]. Given the rapid expansion of the broad corpus of medical literature, LLMs may play an important role in enabling the generation of up-to-date and patient-relevant guidelines.

Furthermore, the flexibility of the transformer architecture means that combining models across modalities can create a powerful multimodal model capable of leveraging information from several contexts, including radiological images, patient notes, lab tests, and genomics data for example [8–10]. These models have already shown impressive capabilities in the generation of radiology reports as well as prediction of clinical diagnosis and patient outcomes [11–13]. As the increasing digitization of health systems enables the greater linking of Electronic Medical Record (EMR) data, multimodal models may play an increasing role to facilitate greater contextualised predictions.

### Understanding the risks of language models in healthcare

However, despite the promise of machine learning in healthcare (MLHC) broadly, there are also significant risks of patient harm. Many models—including those which have been implemented in clinical practice [14, 15]—have been demonstrated to function poorly for minority patient populations. Controversy also exists regarding the “explainability” of such models, and the difficulty in clearly examining the processes which lead to model decisions [16].

There are also risks specific to the LLM architecture and the primacy of text [17]. Firstly, such models have no robust causal models of the world, given that they rely primarily on associative means. LLMs are also prone to so-called “hallucinations”, wherein plausible-sounding but incorrect results are generated with apparent confidence [18, 19], including at times the generation and citation of completely fabricated scientific literature.

Further, LLM models are generated based on broad corpora of unselected text which reflect past and present disparities and biases. For instance, pre-trained text embedding models have been shown to offer differential performance on characteristics such as sex and race, predicting that a belligerent white patient is “sent to *hospital*” while a belligerent African American patient is “sent to *prison*” [20].

## Section 1: the changing role of clinicians

As stated in the popular maxim of Amara’s law, there is a tendency to overestimate the impact of a technology in the short term, and underestimate it in the longer term [22]. Deep learning pioneer Geoffrey Hinton famously opined in 2016 that “We should stop training radiologists now. It’s just completely obvious that within five years, deep learning is going to do better than radiologists” [23]. At the seven year mark, this prediction has yet to come to fruition. Other commentators have predicted the relationship between clinicians and AI to be one of augmentation rather than replacement [24, 25], and experimental evidence has already demonstrated opportunities for collaboration [26]. Similar considerations are likely to apply in the case of LLMs, where achieving potential while minimizing risk will require not only modification of the technology, but also adaptations on the part of clinician users.

Shifts in necessary clinician skills and priorities have always occurred alongside technological changes. For example, the ubiquity and ease of access to bedside reference resources, such as UpToDate or DynaMed, has already begun to reduce the relative value of rote memorization [27, 28]. In its place, however, the ability to rapidly read and summarize literature has grown in prominence with the proliferation of clinical trials and the web search technology. As discussed, the wide-ranging nature of these models is likely to rearrange the importance of a wide range of clinician skills.

Further, the power of this technology is such that the ideal frame of understanding may shift from seeing models in terms of their usefulness to clinicians, to seeing both clinicians and models in terms of their usefulness to the overall medical system. This concept has been explored in other safety-critical domains such as aviation, regarding human and machine components as cooperative elements of a shared system [29, 30]. While human actors in these domains have advantages over their machine counterparts such as creativity in novel situations, they also have relative deficits such as fatigability and cognitive bias. An ideal system is crafted to ensure complementarity of both sets of capabilities.

Given the rapid pace of change and advancement of this technology, flexibility is likely to be a core virtue on the part of providers. Depending on the rapidity of progress, shifts in the use of these technologies may occur every few years. Therefore, providers must be able and willing to evolve in their practice alongside the evolution of technology, while simultaneously demanding rigorous evaluation of these technologies to determine the robustness and utility of their applications. Clinicians, and their organizations, will be called upon to discern which technologies on the market meet their specific needs, while working with limited resources.

The role of clinicians as repositories for medical knowledge may decrease, as such information becomes increasingly accurately embedded in, and easily accessed from online sources and LLM models. At the same time, the evaluator role of clinicians in medical decision making is going to increase, as they are called upon to evaluate and integrate information offered by models in specific clinical contexts. Clinicians must apply their professional judgment in evaluating model outputs, and use the information as an aid to decision-making rather than a replacement for robust reasoning. Clinicians must also understand the limitations of these technologies with respect to their specific patient population. However, it is important to ensure that these technologies do not lead to an abdication of responsibility, and clinicians are held responsible for their endorsement of decisions facilitated by clinical algorithms.

Similar considerations apply to the generation and application of clinical documentation. AI assistance may lead to less time and energy being spent on the summarization of visits, the generation of documents, and the completion of administrative tasks [31]. But it must be understood that summarization remains a critical element of documentation that has legal and ethical implications, and must not be assumed to be a simple, low-risk space for LLM implementation. Clinical documentation plays an important legal role, and clinicians must accept their legal and ethical responsibility for the comprehensiveness and accuracy of documentation written and signed.

In addition, the empathic role of clinicians in understanding a patient’s personality and values will remain essential. Every patient’s health and experience of illness is influenced by complicated biological, psychological, and social contexts which cannot be fully reduced through associative summarization. The information returned by LLMs must be carefully contextualized, with the recognition that an “ideal treatment course” may vary substantially based on the idiosyncratic values and preferences of an individual patient. Clinicians must be trusted to act as strong advocates on the part of patients, ensuring that the use of these models remains grounded in foundational principles of medical ethics, and a shared sense of understanding [21, 32, 33]. On the efficiency front, it should be noted that LLMs are able to increase physician engagement with patients only if physicians are not pressured to translate increased efficiency into higher overall output, for example, seeing more patients within a shorter span of time.

Discernment should also be exercised in the study and development of LLMs for clinical use, ensuring that risks are mitigated and ethics are embedded into the models themselves. We must thus seek to train specialized clinician-scientists and leaders who will play an important role in the design, evaluation, and implementation of any LLM-based technologies in healthcare. Given the broad degree of hype, the technology is at risk of being a “solution in search of a problem”, used in clinical circumstances for which it is not appropriate or optimal. Thus, judicious development and application of LLMs should be practiced.

Any machine learning models used in healthcare require careful ongoing monitoring due to the challenge of “dataset shift”, and the concern that the performance and accuracy of model outputs will decrease over time based on changes in the underlying clinical reality [34]. In addition, the well-known risks related to bias and inequity must be understood, and addressed as foundational challenges rather than secondary afterthoughts [32]. Clinician leaders must work to establish organizational administrative processes in order to satisfy these requirements, and must not abdicate this responsibility to those more distant from patient concerns. Similar concerns apply to the need for development of appropriate regulatory frameworks [35].

## Section 2: what all clinicians must know, and what AI specialists should know

As the body of medical knowledge has grown over the past centuries, so too has the demand for specialization. However, medical students do not train exclusively in one field; they rotate through each specialty, gaining an understanding of each area. The expectation is that clinicians gain an appreciation of the remit of each specialty, an awareness of key emergencies/red flags, and knowledge regarding when to seek specialist advice. A similar dynamic will likely play out with AI, with the dual requirement for developing a baseline of understanding for all clinicians, as well as a more specific body of knowledge for specialists in the field [36].

**Table 1:** Selected competencies for General and AI specialist-clinicians

	General Clinician	AI Specialist-Clinician
Health Informatics Competencies	- Knowledge of data stewardship and patient privacy concerns. - Ability to work with electronic medical records in recording and accessing patient information. - Ability to engage with telemedicine systems. - Awareness of the limitations of health data systems with respect to completeness and representativeness of data. - Ability to adapt to and work with novel informatics interfaces and computer systems. - Ability to use basic clinical decision support systems.	- Detailed understanding of clinical workflows, with the ability to appropriately design models to support given clinical use cases. - Understanding of the social, economic, and political context of AI at the level of health research, health system structure, and health technology regulation. - Organizational management skills to guide informatics implementation projects and workflows. - Proficiency in data management skills, such as data cleaning and quality assurance - Understanding of and ability to align work with common data interoperability standards.
Artificial Intelligence Competencies	- Understanding the applicability of a given AI technology in specific clinical contexts. - Interpreting and explaining the output of a given AI model (or, in the case of unexplainable models, evaluating the empirical validation process of a given model). - Knowledge of the limitations of a given AI model, with a particular emphasis on fairness and bias as well as differential model performance. - Understanding the importance of human oversight and the limitations and failure cases of AI systems. - Ability to recognize and mitigate AI failures as they arise in a clinical workflow.	- Detailed knowledge of model architecture, with assessments of the appropriateness of a specific technology for a given clinical task. - Evaluating the performance and robustness of an AI model for a specific clinical problem. - Evidence-based evaluation of AI-based tools, including trial design, implementation, and continuous monitoring. - Generating and curating datasets for the purpose of - Identifying limitations and biases in performance of algorithms towards marginalized groups and fine-tuning model performances by curating more representative datasets. - Ability to interpret and communicate model performance metrics to non-technical stakeholders. - Awareness of the legal and regulatory landscape for AI in healthcare, including liability concerns and approval processes.
Generative AI / LLM Competencies	- Baseline awareness of the inputs and architectures of LLMs. - Skills in offering both initial and follow-up queries to LLM systems as appropriate in a given clinical context. - Skills in prompt engineering, with awareness of the context-specificity and stochasticity of LLM outputs. - Understanding of the “hallucination” phenomenon, and ability to be appropriately skeptical and verify LLM outputs where necessary. - Integrating information from a diverse range of sources (including LLM summaries or differential diagnostic predictions alongside traditional info such as patient demographics, clinical presentation, and investigation results) in the context of a patient-centered clinical encounter.	- Collaboration with colleagues from other medical specialties to identify opportunity and limitations for further development of LLMs within clinical contexts. - Ability to generate and incorporate human feedback and clinician / patient preference information in fine-tuning models. - Detailed generation and evaluation of prompts, alongside sensitivity analysis for the impact of subtle prompt fluctuations on outputs - Understanding of cutting-edge technical developments in generative AI (such as retrieval-augmented generation (RAG) or long context window techniques). - Ability to design and implement human evaluation studies to assess clinical impacts of LLM-generated content. - Understanding of the ethical and legal implications of using generative AI, including with respect to intellectual property, liability, and privacy.

As the physiological increasingly turns computational, it is easy to mistake the observed for the real, and decision support for dogma. All clinicians should have a baseline set of competencies, including knowledge of (1) forms of bias that can occur in clinical data, (2) uncertainty of predictions, (3) causal inference and confounding. Clinicians must have an understanding of how these systems apply to their individual patients, with particular emphasis on issues of equity and an awareness that such systems do not perform equally for all. For an illustrative example, generalists require only a cursory awareness of the particle spin physics underlying MRI scans, but must have a clear understanding of when an MRI should be ordered for a given patient, how to interpret and explain its results in a clinical context, and when an MRI may be of limited or even misleading utility.

In addition to these baseline competencies, we must seek to train AI Specialist-Clinicians with a combined understanding of medicine and computer science, able to play important roles in the development and implementation of these models [37]. Such clinicians will require a much deeper understanding of fundamental model architecture, and the capabilities and limitations of a given approach. In addition to their work in model development and monitoring, we envision such clinicians being able to offer specific consultations on AI-related questions. An instructive analogy here would be the role of a radiologist in helping to guide appropriate imaging modality selection, and discussing the implications of unclear or unexpected results.

## Section 3: situating LLM skills in the broader context

The skills necessary to take advantage of LLMs do not exist in isolation. Rather, they must be understood in the context of a long history of foundational work on competencies in health informatics [38] as well as more recent work on AI broadly [36]. Rather than bypassing these foundations and “reinventing the wheel”, LLM-specific education must be complementary. As summarized in Table 1, both the general clinician and the AI specialist clinician require skills in all three domains in order to find success.

Whether in sourcing the data for their inputs, or communicating their outputs to clinicians, many modern LLM-based systems are being constructed in tandem with EMRs [39] and their underlying databases. While there is some prospect of utilizing LLMs themselves to improve the quality and structure of EMR data [39, 40], many of the existing challenges with medical data persist and indeed may only grow in importance alongside the increasing power of medical AI. Ability to efficiently and effectively use these systems while safeguarding patient privacy and interests is important to every physician. Ai specialist leaders must be able to go further, and effectively lead the organizational transitions necessary for health data to contribute to care-enhancing systems in real time.

With AI systems moving from merely providing information to providing recommendations and more specific judgments, the skills required of practitioners grow in specificity [36]. This entails knowledge of the specific strengths and weaknesses of such systems, with a particular emphasis upon the cases where they may underperform or outright fail. While the general clinician must learn to appropriately engage with information and recognize such failures, the AI specialist clinician must be able to go further, designing and evaluating systems to mitigate failures in the first place. AI specialists must also be able to navigate the complicated social, legal, and ethical implications of these technologies, and translate between technical and non-technical stakeholders in both development and implementation.

Engagement with generative AI — and LLMs specifically — requires these skills to be nuanced and focused upon the particular strengths and limitations of this nascent technology. The novel skill of “prompt engineering” (that is, designing text inputs to achieve the appropriate outputs) is necessary both at the level of generalists and AI specialists [41]. While the former must be able to effectively prompt in the context of a clinical workflow, the latter must be able to systematically analyze and optimize prompting techniques (which, as recent research has shown [42], can vastly improve model performance). LLMs are also unique in their ability to be confidently persuasive while “hallucinating” [43], necessitating a degree of appropriate skepticism from clinicians, and the ability to verify information given. With the field so rapidly evolving, it is important for AI specialist clinicians to keep abreast of the technical detail of new generative AI research, and connect it effectively to medical practice.

## Section 4: required adaptations of medical education

Despite the increasing interest in artificial intelligence in medicine, existing approaches to medical education on the subject are inconsistently offered, and have highly variable content between centers [44]. LLM-specific education is relatively nonexistent at this time. We believe that the tremendous rate of advancement in these technologies necessitates significant ongoing investment in addressing medical education challenges in this regard. Students entering medical school in 2024 will enter independent practice in the early- to mid-2030s, to say nothing of the training that existing clinicians will require. Training must be forward-looking, and early steps must be taken with urgency to ensure that the medical system is up to the task [45].

First is the question of “what”. There is little agreement regarding what core competencies must be taught to medical students regarding this topic, with major differences between institutions such as Harvard University, and the University of Toronto [36, 46]. Broad, multi-stakeholder efforts are required to establish shared competency frameworks and enable collaboration to develop collective resources [47]. This may take the form of a specific analogue to existing efforts such as the development of the CanMEDS framework, outlining multiple domains of broad clinician competency [48]. In addition, there must be a core recognition (as we have outlined above) of the difference between what *all* must know and what must be done to train clinical AI specialists [36, 49].

Second is the question of “who”. The interdisciplinary nature of AI technologies requires the insight and expertise not only of clinicians, medical educators, and their existing primary collaborators in pre-clinical departments, such as anatomy and physiology. On the technical side, expertise is needed from computer scientists, engineers, cybersecurity experts, and bioinformaticians among others. Further, in order to understand the context and risks of these models, input is required from ethicists, sociologists, medical anthropologists, and others. The intensely complicated and multi-disciplinary nature of this technology will necessitate the creation of new professional relationships and organizations cutting across these divides. This will require upstream adaptation of existing hierarchies and recruitment pathways in medical schools, in order to create a robust and responsive faculty base.

Third is the question of “how”. Given how little consensus exists regarding what must be taught [50], it is unsurprising that there is little consensus regarding the optimal methods of AI education. We believe that the recent progress has significant implications not only for AI-specific education, but also for medical education more broadly. Progress in this field must prompt a more radical reconceptualization of medical education broadly. The relative ease with which these models are able to pass the USMLE examination, for example, may call into question the structure of this examination process, and the significant portion of study time which is spent on the rote memorization of facts. With wide bodies of factual information at the fingertips of clinicians, we posit that there must be a broad shift from an emphasis on *knowledge* to an emphasis on *skills*. It is encouraging that over the last year, the grading of the USMLE exam has moved to Pass/Fail in place of a numeric score [51].

Concurrently, we advocate for a transition away from the more traditional medical education model to a pathway-driven model in which learners can leave medical school with a deeper area of expertise in a chosen domain (e.g. medical computing, public policy/social medicine, clinical research, etc.). In this way, medical education better mirrors other professional schools such as business, law, and engineering and would provide the necessary time to specialize in important contemporary topics, such as AI and LLMs. Similarly, as students use their foundational medical training to determine their field of practice, they will also have the opportunity to hone non-clinical skills and knowledge and discover ways to impact medicine on a larger scale.

Attention in this regard must not be solely focused at the undergraduate and postgraduate medical education levels. The majority of users of LLM based technology, if it is implemented in healthcare within the next decade, will be existing clinicians who have received little to no specific training in the topic. Efforts must be made on the continuing medical education front to prepare clinicians to effectively use these technologies. Of equal importance will be research and examination of existing healthcare processes on the front of AI technology, and to ensure safe and effective implementation.

It is also crucial to acknowledge the contributions of established educational theory in this process. Constructivist theory, in particular, aligns well with the challenges of teaching AI skills in a rapidly evolving field. This theory emphasizes that learners actively construct knowledge through experiences and interactions with their environment, rather than passively receiving information [52]. In the context of AI education for medical professionals, constructivist approaches would involve experiential, active learning within clinical care settings. This is especially relevant as both learners (medical students and residents) and teachers (supervising physicians) may be simultaneously developing their AI competencies. Problem-based learning, case studies, and hands-on projects involving real-world AI applications in healthcare could be effective strategies rooted in constructivist theory. This approach not only helps in skill development but also cultivates critical thinking and adaptability – crucial attributes for navigating the dynamic landscape of AI in medicine.

Ultimately, changes to the how, who and what of medical education should also reflect on grading and testing practices. As LLMs have repetitively been shown to easily pass standardized tests, such as USMLE, this raises the urgent question if a transition away from knowledge to understanding, reasoning and critical thinking is needed [53]. 

## Conclusion

Recent advances in LLM technology have been striking in both their magnitude and pace, raising the prospect for a future where such technologies are deeply integrated into clinical practice. These developments bring the opportunity for improving the quality of care through rapid summarization and presentation of medical knowledge, as well as significantly reducing administrative burden. At the same time, there are significant risks associated with these models, particularly in relation to datasets embedding existing societal biases, and the specific tendency of LLMs toward overconfident “hallucination” of answers.

Nonetheless, these systems are primed to have a wide ranging series of impacts on healthcare, and all clinicians must be effectively trained to take advantage of these opportunities while countering the associated risks. This task will require a collective undertaking toward understanding the “what”, “who”, and “how” of AI-related medical education, in order both to establish a baseline level of competence among clinicians broadly, and to facilitate the training of clinicians with a deeper level of AI-specific expertise. Medical education for students and practicing clinicians alike must adapt with rapidity and dynamism matching the pace of technological change.

## Data Availability

No datasets were generated or analysed during the current study.

## Abbreviations

LLM: Large Language Model
MLHC: Machine Learning for Healthcare
USMLE: United States Medical Licensing Examinations
AI: Artificial Intelligence
EMR: Electronic Medical Record
